# Economic evaluation of facility‐based HIV self‐testing among adult outpatients in Malawi

**DOI:** 10.1002/jia2.25612

**Published:** 2020-09-09

**Authors:** Brooke E Nichols, O Agatha Offorjebe, Refiloe Cele, Frackson Shaba, Kelvin Balakasi, Mackenzie Chivwara, Risa M Hoffman, Lawrence C Long, Sydney Rosen, Kathryn Dovel

**Affiliations:** ^1^ Department of Global Health School of Public Health Boston University Boston MA USA; ^2^ Health Economics and Epidemiology Research Office Department of Internal Medicine School of Clinical Medicine Faculty of Health Sciences University of the Witwatersrand Johannesburg South Africa; ^3^ David Geffen School of Medicine University of California Los Angeles Los Angeles CA USA; ^4^ Charles R. Drew University of Medicine and Science Los Angeles CA USA; ^5^ Partners in Hope Lilongwe Malawi; ^6^ Division of Infectious Diseases Department of Medicine University of California Los Angeles Los Angeles CA USA

**Keywords:** cost‐effectiveness, health systems, modelling, testing, HIV self‐testing, economic evaluation

## Abstract

**Introduction:**

HIV self‐testing (HIVST) in outpatient departments (OPD) is a promising strategy for HIV testing in Malawi, given high OPD patient volumes and substantial wait times. To evaluate the relative cost and expected impact of facility‐based HIVST (FB‐HIVST) at OPDs in Malawi for increasing HIV status awareness, we conducted an economic evaluation of an HIVST cluster‐randomized controlled trial.

**Methods:**

A cluster‐randomized trial was conducted at 15 sites in Malawi from September 2017 to February 2018 with three arms: 1) Standard provider‐initiated‐testing‐and‐counselling (PITC); 2) Optimized PITC (additional provider training and job‐aids) and 3) FB‐HIVST (HIVST demonstration, distribution and kit use in OPD, private kit interpretation and optional HIV counselling). The total production cost per newly identified positive and per person newly initiated on ART were calculated by study arm. These were calculated as the total cost of testing everyone divided by the number of newly identified positives; and the total cost of testing everyone divided by the number of those initiated on ART. Cost‐outcomes were calculated under three cost scenarios: (1) full study costs, (2) routine implementation costs and (3) routine implementation + reduced cost for HIVST kits.

**Results:**

The average cost per person newly diagnosed in the full study cost scenario was $101, $156 and $189, and cost per person initiated on ART was $121, $156 and $279 for Standard PITC, Optimized PITC and FB‐HIVST respectively. In the routine implementation cost scenario, the average cost per person newly diagnosed was reduced to $83, and $93, and cost per person initiated on ART to $83, and $137 for Optimized PITC and FB‐HIVST respectively. In the negotiated HIVST cost scenario, the average cost per person newly diagnosed was reduced to $55 and cost per person newly initiated on ART reduced to $81 in the FB‐HIVST arm.

**Conclusions:**

While the cost per new ART initiation through FB‐HIVST was higher than Standard PITC, FB‐HIVST could become cost‐saving compared to PITC if the cost of kits is reduced or if treatment linkage rate were increased in the FB‐HIVST arm. For high volume OPDs, HIVST may increase facility capacity and increase the number of newly diagnosed positives.

## 1 INTRODUCTION

To help national human immunodeficiency virus (HIV) programmes achieve the global goal of ending the acquired immunodeficiency syndrome (AIDS) epidemic by 2030, UNAIDS has set ambitious targets known as “95‐95‐95”: 95% of people infected aware of their status, 95% of those aware on treatment and 95% of those on treatment achieving a suppressed viral load [1]. While great strides have been made in expanding antiretroviral therapy (ART) coverage and achieving viral suppression, HIV case finding is lagging behind [2]. In Malawi, a high‐prevalence country in southern Africa, 30% of people who are HIV‐positive remains unaware of their status [2, 3].

HIV self‐testing (HIVST) is recognized as an innovative method to increase the proportion of people who know their HIV status [4, 5]. Malawi has already adopted oral swab and oral fluid HIVST into its national HIV treatment and prevention strategy [6], and both the accuracy and acceptability of HIVST have been established within community‐based settings [7, 8, 9]. Nationwide distribution of HIVST kits in Malawi, however, will largely depend on cost and expected impact compared to the current standard of care. The cost of current HIVST delivery strategies has been found to be higher than routine facility‐based HIV testing using rapid, finger stick test with whole blood, mainly driven by HIVST kit cost and cost of community‐based distribution [10]. The distribution cost could be reduced with changes to the HIVST delivery approach [4, 5, 11]. Facility‐based distribution of HIVST kits is one such approach and has been shown to increase HTC uptake compared to provider‐initiated testing and counselling (PITC) [5, 12]. In Malawi, previous HIVST economic evaluations have primarily assessed costs associated with home‐based HIVST delivery [10, 13]. So far, none have conducted an economic evaluation of delivering HIVST to patients seeking routine healthcare services at facilities.

To evaluate the relative cost and expected impact of HIVST at outpatient departments (OPD) in Malawi for increasing HIV status awareness, we conducted an economic evaluation of an HIVST cluster‐randomized controlled trial [12, 14].

## 2 METHODS

### 2.1 HIVST trial in the outpatient department

The HIVST study was a cluster‐randomized controlled trial that examined the integration of HIVST into OPDs in health facilities in Malawi from September 2017 to February 2018 [12, 14]. In Malawi, clients wait for outpatient services an average of four hours [15], providing an opportunity for HIVST while waiting to see a provider. The trial compared HIV testing uptake and positivity rates (or number of HIV positives divided by total tested) between three study arms across 15 facilities (facilities randomized 1:1:1). All arms included some variation of opt‐out testing offered by healthcare workers or study staff. In Arm 1, Standard PITC, providers could refer outpatients to the HIV department for testing (non‐integrated service delivery). In Arm 2, Optimized PITC, HTC staff received additional training on PITC and HIV testing was provided in the morning in OPD before routine outpatient services were offered, allowing for integrated service delivery. In Arm 3, Facility‐based HIVST (FB‐HIVST), HIVST kits were offered to outpatients, used and interpreted before they were seen for routine outpatient services. Outpatients were strongly encouraged to use HIVST kits at OPD, and not take the kits home. Private booths were placed in or outside the back of the OPD waiting rooms for test interpretation. Since self‐testing was performed at the facility, individuals who screened positive and chose to disclose their test results to providers could be referred directly to facility counsellors for test confirmation and linkage to care.

Primary outcomes were measured with a one‐time, anonymous exit survey conducted with participants aged 15+ years after outpatient and HIV services were received. Eligible individuals gave oral consent. Written informed consent was obtained from those individuals who reported testing HIV positive on the day of enrolment for purposes of chart review to determine whether or not participants initiated ART within three months of study enrolment.

### 2.2 Costing and cost scenario analysis

Costs for the observed HTC algorithm from five facilities were captured using a micro‐costing (bottom‐up) approach from the provider perspective [16]. These facilities were representative of the main types of health facilities in Malawi, including government and Christian mission health facilities of different sizes. The additional cost of providing optimized PITC and FB‐HIVST were sourced from study records and expenditure reports [12]. No additional costs related to supply chain were included, as we assumed standard supply chain mechanisms, given that cold‐chain is not required. The cost of HIV testing service provision was calculated per individual tested, including the cost of testing negative and/or positive. In the HIVST arm, this included the full facility‐based testing algorithm after a positive HIVST screen.

Three cost scenarios were evaluated. In Scenario 1, *Study Cost Scenario*, we estimated base case unit costs by study arm using the trial‐based cost parameters. Full training costs, staff salaries and community sensitization costs in the Optimized PITC and HIVST scenarios were allocated across the number tested in each arm. These costs, and the cost of Standard PITC by facility have been previously reported, and are reported here for purposes of comparison [12].

In Scenario 2, *Routine Implementation Cost Scenario,* we reduced implementation costs in the Optimized PITC and FB‐HIVST arms to mimic “real world” implementation. Staff training costs were assumed to be repeated every two years, rather than being fully allocated to those tested in the study period (two to six weeks). We assumed that community sensitization costs in the implementation of HIVST would occur annually. Finally, we replaced the study staff salaries with Malawian Ministry of Health salaries, as government staff would be distributing and demonstrating the use of HIVST kits in a practice.

Finally, in Scenario 3, *Negotiated HIVST Cost Scenario,* we explored the impact of reducing a HIVST kit price to the price of the most expensive standard HIV test kit in Malawi, $1. This is the price of a rapid, finger stick test with whole blood. For this scenario, routine implementation costs from Scenario 2 were utilized.

Costs were collected in Malawian Kwacha then converted to US Dollars using exchange rate of MKW 718.92 to 1 USD (exchange rate averaged from May to December 2017) [17]. Costs are reported in 2017 US dollars.

### 2.3 Cost‐outcomes analysis

The total production cost per newly identified positive and total cost per person newly initiated on ART were calculated by study arm. These were calculated as the total cost of testing everyone (positive and negative) divided by the number of newly identified positives; and the total cost of testing everyone divided by the number of those initiated on ART. The costs in the numerator were then varied by the three cost scenarios. This analysis was further stratified by sex.

### 2.4 Sensitivity analysis

Routine implementation of FB‐HIVST may affect results, such that while costs may decrease, so may expected outcomes. The HIV testing yield may also decrease over time within a testing programme with very high testing uptake such as FB‐HIVST. We therefore conducted a threshold analysis to assess by what percent HIV testing yield and rate of initiation on ART could decline and remain cost‐saving or cost‐neutral in the Routine Implementation Cost and Negotiated HIVST Cost scenarios [16].

The underlying testing yield, which directly affect the production cost of a newly identified positive, naturally differ by site. To assess the impact of testing yield on outcomes, we held the positivity rate constant at 2.7% across all three arms in a sensitivity analysis and assessed how that affected production costs.

### 2.5 Cost and impact of national scale‐up

Using data from the original trial, alongside national HIV testing data, we estimated the total expected number of new HIV diagnoses and related costs through the addition of FB‐HIVST to Standard PITC. First, in the five sites that offered FB‐HIVST, average Standard PITC percent positivity in 2017 was compared, by sex and facility type, to the percent positivity found with HIVST in the OPD during the study period. These differences in positivity as compared to Standard PITC positivity, by site type and sex, were then applied to the number tested through Standard PITC at all 652 public healthcare facilities from October 2016 to September 2017. We stratified scale‐up by facility‐type and ensured that the increase in uptake was proportional to facility size and current Standard PITC numbers. Second, it is possible that introduction of HIVST to the OPD would reduce Standard PITC at that facility. In order to assess whether HIVST through the OPD would either (1) have an additive effect or (2) displace some Standard PITC numbers tested, we calculated the number tested through Standard PITC at FB‐HIVST sites during the HIVST study period. To calculate the additive or displacing effect of FB‐HIVST, the total number of people tested through the OPD at the FB‐HIVST sites was then related to the total number of people tested, by site type and sex, through Standard PITC during the same time period. The main trial was not powered to detect combined facility‐level and sex differences between Optimized and Standard PITC. As such, scale‐up of Optimized PITC could not be modelled.

Costs for the national estimates were taken from our second cost scenario, “routine implementation costs.” The cost per test expected with routine implementation was multiplied by the expected number of tests to determine the total cost to scale‐up.

### 2.6 Ethics

The original trial and related economic evaluation were approved by the institutional review board (IRB) of the University of California, Los Angeles and the Malawian National Health Sciences Research Committee.

## 3 RESULTS

During the study period, 1572 OPD patients completed an HIV test. Standard PITC (arm 1) had the lowest uptake at 13% (248/1,951 outpatients seen) and lowest rate of newly identified positives (2.4%, 6/248) (Table 1). Optimized PITC (arm 2) had similar uptake at 14% (261/1,837), and a positivity rate of 3.1% (8/261). FB‐HIVST (arm 3) had the highest uptake, with 51% (1,063/2,097) of patients eligible accepting a self‐test, and a 2.6% positivity rate (28/1063).

**Table 1:** Primary HIV self‐test cluster‐randomized controlled trial at the Malawian outpatient department: outcome data

		Arm	
	Standard PITC	Optimized PITC	FB‐HIVST
Study outcomes			
Total			
Tested	248	261	1063
Newly identified positive (% newly identified of total)	6 (2.4%)	8 (3.1%)	28 (2.6%)
Initiated on ART (% initiated of total)	5 (2.0%)	8 (3.1%)	19 (1.8%)
Adult men (age 25+)			
Tested	57	87	221
Newly identified positive (% newly identified of total)	3 (5.3%)	5 (5.7%)	9 (4.1%)
Initiated on ART (% initiated of total)	3 (5.3%)	5 (5.7%)	7 (3.2%)
Adult women (age 25+)			
Tested	105	78	450
Newly identified positive (% newly identified of total)	2 (1.9%)	2 (2.3%)	11 (2.4%)
Initiated on ART (% initiated of total)	1 (1.0%)	2 (2.3%)	9 (2.0%)
Adolescents			
Tested	86	94	381
Newly identified positive (% newly identified of total)	1 (1.2%)	1 (1.1%)	8 (2.1%)
Initiated on ART (% initiated of total)	1 (1.2%)	1 (1.1%)	3 (0.8%)

Positivity rates were generally higher amongst adult men, at 5.3%, 5.7% and 4.1% in the Standard PITC, Optimized PITC and FB‐HIVST arm respectively. For adult women, there was a 1.9%, 2.3% and 2.4% positivity in the Standard PITC, Optimized PITC and FB‐HIVST arm respectively. Positivity was lowest among adolescents (1.2%, 1.1% and 2.1% in the three arms respectively) [12].

Within three months of testing positive, 83% (5 out of 6) of newly identified positives initiated ART in the Standard PITC arm, 100% (8/8) in the Optimized PITC arm and 68% (19/28) in the FB‐HIVST arm.

### 3.1 Cost per person tested

The cost per person tested under Standard PITC was $2.44 (Table 2). The main components of this cost were consumables (57%), followed by staff time (38%). Of the consumables cost, 60% was for the HIV test kit, at between $0.80 and $1.00 per test. In the Study Cost scenario, the cost per person tested for HIV test under Optimized PITC was $4.79, and fell to $2.53 in the Routine Implementation Cost scenario. The cost per person tested for HIV under HIVST was $4.99, $2.45 and $1.45 in the Study, Routine Implementation and Negotiated HIVST Cost scenarios respectively.

**Table 2:** Cost per person tested by cost category (2017 USD)

	Staff^a^	Equipment^b^	Consumables^c^	Facility overheads^d^	Training	Community sensitization	Total
Study costs							
Standard PITC	$0.95	$0.08	$1.37	$0.03	$0.01	$0.00	$2.44
Optimized‐PITC	$0.95	$0.08	$1.37	$0.03	$2.36	$0.00	$4.79
FB‐HIVST	$1.22	$0.26	$2.07	$0.00	$0.31	$1.14	$4.99
Routine implementation costs							
Standard PITC	$0.95	$0.08	$1.37	$0.03	$0.01	$0.00	$2.44
Optimized‐PITC	$0.95	$0.08	$1.37	$0.03	$0.10	$0.00	$2.53
FB‐HIVST	$0.31	$0.01	$2.07	$0.00	$0.01	$0.04	$2.45
Negotiated HIVST cost							
Standard PITC	$0.95	$0.08	$1.37	$0.03	$0.01	$0.00	$2.44
Optimized‐PITC	$0.95	$0.08	$1.37	$0.03	$0.10	$0.00	$2.53
FB‐HIVST	$0.31	$0.01	$1.07	$0.00	$0.01	$0.04	$1.45

^a^ Depending on group, costs include HIV counsellors, outpatient department providers and study staff who conducted the HIV self‐testing intervention

^b^ includes all facility‐based equipment for standard and optimized provider‐initiated testing and counselling, including testing booths for those in the HIV self‐test group and facility‐based equipment for those who were screened positive and went through the testing algorithm at the facility

^c^ depending on group, costs include HIV self‐testing kits and standard‐of‐care testing supplies

^d^ includes building maintenance, utilities and building infrastructure.

### 3.2 Cost per newly identified positive

**Figure 1:**
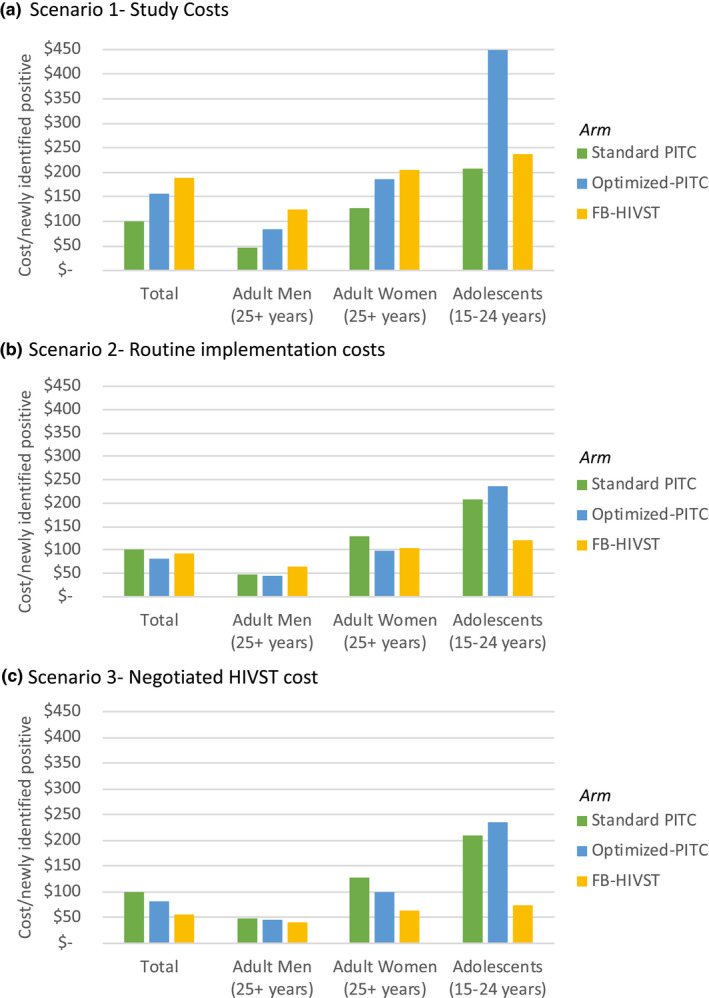
Cost per newly identified positive, total and by age group, by cost scenario (A‐C).(A) Scenario 1‐ Study Costs. (B) Scenario 2‐ Routine implementation costs. (C) Scenario 3‐ Negotiated HIVST cost.

In the Study Cost scenario, the average cost per newly identified positive was $101, $156 and $189 in the Standard PITC, Optimized PITC and FB‐HIVST arm respectively (Figure 1). Given the higher yield among adult men, the cost per newly identified positive in the FB‐HIVST arm was 65% more for adult women ($204), and 91% more for adolescents ($237) as compared to adult men ($124).

In the Routine Implementation Cost scenario, the cost per new positive identified decreased to $83 and $93 in the Optimized PITC arm and FB‐HIVST arm respectively. In the Negotiated HIVST Cost Scenario, the cost per newly identified positive in the FB‐HIVST arm decreased 41% to $55.

#### 3.2.1 Sensitivity analysis: newly identified positives

In the Negotiated HIVST Cost Scenario, if the HIV test yield was reduced by ≤45% for FB‐HIVST and held constant for Standard PITC, FB‐HIVST would cost less per newly identified positive compared to Standard PITC. If the HIV test yield was reduced by ≤33% and held constant for Optimized‐PITC, FB‐HIVST would cost less per newly identified positive as compared to Optimized PITC.

If the HIV testing yield remained constant at 2.7% across arms, in the Routine Implementation Cost scenario, the cost per newly identified positive was nearly identical between Standard PITC and FB‐HIVST ($90.37 and $90.74 respectively), and highest in Optimized‐PITC ($93.70). In the Negotiated HIVST Cost scenario, the cost of FB‐HIVST further reduced to $53.70 per newly identified positive.

### 3.3 Cost per patient initiated on ART

**Figure 2:**
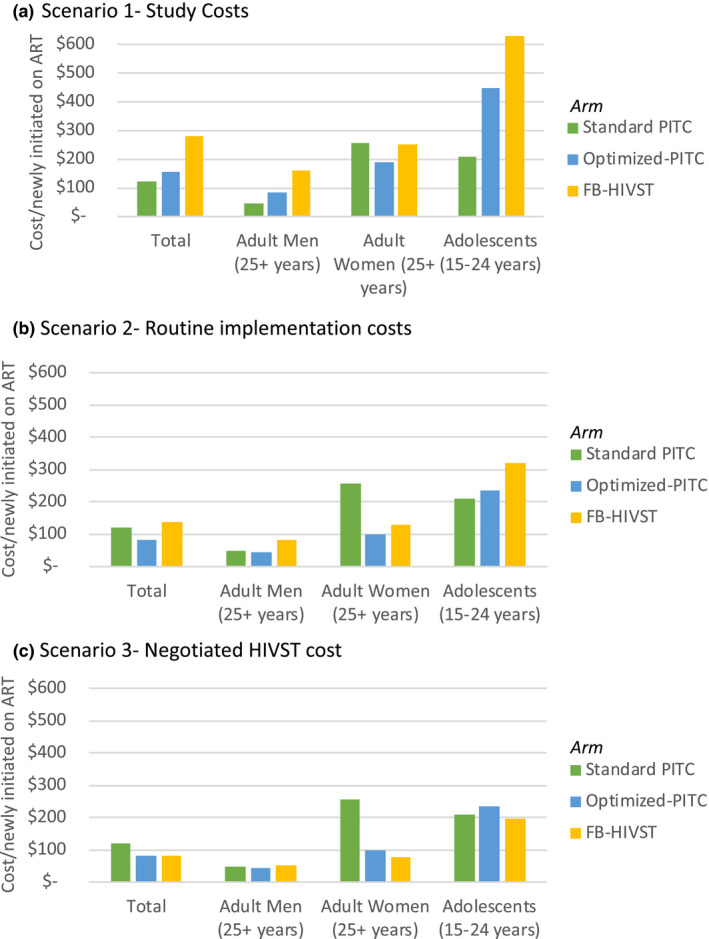
Cost per newly initiated on ART, total and by age group, by cost scenario (A‐C). (A) Scenario 1‐ Study Costs. (B) Scenario 2‐ Routine implementation costs. (C) Scenario 3‐ Negotiated HIVST cost.

In the Study Cost scenario, the average cost per person newly initiated on ART was $121, $156 and $289 in the Standard PITC, Optimized PITC and FB‐HIVST arm respectively (Figure 2). These production costs differ to the cost per new diagnosis due to the differences in ART initiation rates by arm.

In the Routine Implementation Cost Scenario, the cost per person newly initiated on ART decreased to $83 and $137 in the Optimized PITC and FB‐HIVST arm respectively. In the Negotiated HIVST Cost Scenario, the cost per newly initiated on ART in the HIVST arm decreased to $81.

#### 3.3.1 Sensitivity analysis: newly initiated on ART

In the Routine Implementation Cost scenario, Optimized‐PITC would remain cost‐saving compared to Standard PITC with a linkage rate from testing to treatment of 68%. If linkage rates within the FB‐HIVST arm were improved from 68% to 77%, then FB‐HIVST could be considered cost‐neutral in this cost scenario.

In the Negotiated HIVST Cost scenario, FB‐HIVST would remain cost‐saving compared to Standard PITC and Optimized PITC with linkage rates from testing to treatment of 45% and 67% respectively.

### 3.4 Cost and impact of HIVST scale‐up

From October 2016 to September 2017, 146,785 people were diagnosed with HIV in Malawi. An estimated 90,000 tested positive through PITC in public facilities through 2,600,000 tests, representing 61% of all positives identified. The addition of FB‐HIVST to the OPD is expected to increase the numbers of HIV tests performed by 1,200,000 and result in 28,500 new diagnoses, or a 19% increase in new diagnoses nationally (Table 3). The total annual cost of public sector PITC was estimated at $6,146,000 nationally, or $68 per new positive found. The addition of HIVST to the OPD to Standard PITC would cost, assuming routine implementation costs, an additional $2,940,000 annually, or approximately $103 per additional newly diagnosed individual. The marginal cost per newly diagnosed positive is higher for FB‐HIVST than PITC due to an expected lower testing yield for FB‐HIVST and a higher average cost per test for FB‐HIVST as compared to Standard PITC. There was no evidence of a decrease in facility‐level PITC during FB‐HIVST implementation. This suggests that the FB‐HIVST programme would reach patients who might not otherwise test, and that the FB‐HIVST programme would be purely additive to current Standard PITC. If the price of the HIVST could be reduced to $1/test, then the average cost per new person identified would actually decrease to $67 when the two testing strategies are combined.

**Table 3:** Cost and impact of national facility‐based HIVST scale‐up in Malawi

	Standard PITC	Facility‐based HIVST at OPD	Total (FB‐HIVST added to PITC)
Number tested			
Total number annual tests conducted	2,622,000	1,200,000^a^	3,822,000
Number of positives identified	90,000	28,500	118,500
Routine implementation cost scenario			
National annual cost	$6,146,000	$2,940,000	$9,086,000
Cost per newly confirmed positive	$68	$103	$77
Negotiated HIVST cost scenario			
National annual cost	$6,146,000	$1,740,000	$7,886,000
Cost per newly confirmed positive	$68	$61	$67

^a^ Based on the additional volume predicted by HIVST at the OPD.

## 4 DISCUSSION

In this economic evaluation of a cluster‐randomized trial, assuming routine implementation costs, the cost per test completed was similar for FB‐HIVST and Standard PITC, and the cost per person newly identified as HIV positive was lower for FB‐HIVST than Standard PITC. Recent work suggests that testing strategies that cost less than $200 per new diagnosis are very like to be cost‐effective using the traditional metric “cost per DALY averted,” rendering FB‐HIVST likely to be cost‐effective in this analysis across all scenarios [18]. FB‐HIVST also has the potential to reduce the cost and facility burden of testing, thus leading to a reduction in the cost and human resource requirements to find HIV‐positive individuals. The potential reductions in the cost are related to a reduction in staff time and consumables required. Importantly, by reducing staff time required for testing, staff can focus on other activities such as initiating persons newly diagnosed with HIV on ART, providing care for acutely ill patients, and/or providing adherence counselling for those not virally suppressed.

Across the board, FB‐HIVST demonstrates particular efficiency at diagnosing men, resulting in the lowest cost per person newly aware of their status and newly initiated on ART as compared to both women and adolescents. This is due to the relatively higher yield among men. Women may have lower yield through FB‐HIVST due to possible differences in health seeking behaviour as compared to men, and comprehensive testing through prevention of mother‐to‐child‐transmission programmes [19].

When the price of an HIVST kit is reduced from $2 to just $1, FB‐HIVST is considered cost‐saving in our analyses, even when the percentage of those newly initiating ART is reduced by up to 41% as compared to Standard PITC. This is a plausible scenario given that the public sector HIVST kits price in low and middle income countries (LMICs) has already dropped to $2 from a previous price of between $3‐6 [4], and the current price of HIV rapid tests is $0.69‐$1.60 in Malawi and neighbouring countries [10, 20]. Similarly, a simulation analysis suggested that for HIVST to be cost‐effective in a LMIC context, the kit price should be reduced to at least $1.50 [21]. Other studies have concluded that HIVST becomes most cost‐saving in high incidence settings [13, 21].

Cost estimates from our HIVST trial differed from cost estimates in our model of national scale up of HIVST in health facility OPDs: $101 per new positive found for Standard PITC in our study setting, and $68 per new positive found for Standard PITC in our model of national scale‐up. The variation between the trial evaluation and national calculations is due to national composition of site types, sex and current positivity yield. Specifically, the Standard PITC HIV testing yield in our study was 3.9% for all men and 1.8% for all women, and national estimates were 4.3% for all men and 3.0% for all women. The higher national HIV testing yield resulted in a substantially lower cost per new positive for national estimates.

Our per‐client‐tested cost estimates vary slightly from other economic evaluations on HIVST, where HIVST was estimated to cost $8.80 [10]. The difference in these costs are attributable to different modalities of HIVST delivery used, such as community‐based testing [10], or drug store/vending machine distribution [22], which are more costly and result in a lower yield of newly identified positives. To our knowledge, no other study has estimated the cost of HIVST delivered at a facility.

Although the study is the first to conduct an economic evaluation of FB‐HIVST in a LMIC setting, there are several limitations. First, the data related to new initiation on ART was only available for up to three months after HIV testing, and may be incomplete as individuals may have linked to care after three months. Incomplete linkage data would, however, most strongly underestimate the impact of HIVST: the cost per ART initiation in the FB‐HIVST arm would only decrease with more complete linkage data and/or if strategies can be identified to improve linkage rates among those who test via HIVST in OPD. Second, the five HIVST sites may not represent the national OPD settings. It could be that these sites are among those that would benefit the most from FB‐HIVST, and our national estimates may overestimate the expected number of HIVST tests that would be completed in OPD settings. Third, we assume that the uptake of FB‐HIVST will persist after the study period for our national estimates. However, it is possible that the OPD client pool becomes saturated and the number of FB‐HIVST kits completed start to decline. In this case, our national estimates would overestimate the cost and impact of rollout. Fourth, we did not model the potential cost and impact of scaling up Optimized PITC. However, it is unlikely that Optimized PITC would substantially increase new diagnoses given similar uptake rates as Standard PITC. Fifth, the cost per patient initiated on ART results was based on ART initiation rates from the main trial that were very limited, and therefore not precisely measured. We dealt with this as part of our sensitivity analysis surrounding potential differing initiation rates. Finally, we assumed no facility‐level overhead costs for HIVST in the OPD, as we assumed that the people would be occupying the OPD space and using the same resources for their OPD appointment whether or not they chose to utilize HIVST.

## 5 CONCLUSIONS

National implementation of HIVST in OPD clinics in Malawi, in addition to standard PITC, is likely to be effective at identifying more HIV‐positive individuals who would not have otherwise tested, at a modest additional expense. While new diagnosis and ART initiation through FB‐HIVST were slightly more expensive than Standard PITC, HIVST may become cost‐saving in terms of cost per person newly diagnosed if the cost of kits is reduced. While FB‐HIVST could support more rapid achievement of the first 95‐95‐95 target, other approaches that reach people outside facilities, such as high‐risk persons, will likely continue to be necessary as part of a comprehensive testing approach.

## COMPETING INTERESTS

We declare no competing interests.

## AUTHORS’ CONTRIBUTIONS

BEN, RMH, SR and KD conceptualized the study. OAO, RC and FS led cost data collection. KB, MC led study data collection. BEN led data analysis and OAO, RC, KD contributed to data analysis. All authors contributed to data interpretation. BEN and RC wrote the first draft of the manuscript with input from LCL, RMH, SR and KD. OAO, FS, KB and MC critically reviewed and revised the manuscript. All authors critically reviewed a revised draft of the manuscript. All authors have read and approved the final manuscript.

## ABBREVIATIONS

AIDS, acquired immunodeficiency syndrome; FB‐HIVST, facility‐based HIV self‐testing; HIV, human immunodeficiency virus; HIVST, HIV self‐testing; HTC, HIV testing and counselling; LMIC, low‐ and middle‐income country; OPD, outpatient department; PITC, provider‐initiated testing and counselling.
